# Seeing the positive in the face of adversity: neurocognitive markers of risk and resilience in emotional conflict processing in children with maltreatment exposure

**DOI:** 10.1007/s00787-026-03055-x

**Published:** 2026-05-18

**Authors:** Ferdinand Hoffmann, Lea Bentz, Sonja Entringer, Sibylle M. Winter, Claudia Buss, Christine Heim

**Affiliations:** 1 Institute of Medical Psychology, Charité – Universitätsmedizin Berlin, corporate member of Freie Universität Berlin and Humboldt-Universität Zu Berlin, Luisenstr. 57, 10117 Berlin, Germany; 2 Division of Clinical-Psychological Intervention, Department of Education and Psychology, Freie Universität Berlin, Berlin, Germany; 3 German Center for Mental Health (DZPG), Partner Site Berlin, Potsdam, Germany; 4 Department of Child and Adolescent Psychiatry, Psychotherapy and Psychosomatics, Charité – Universitätsmedizin Berlin, corporate member of Freie Universität Berlin and Humboldt-Universität Zu Berlin, Berlin, Germany; 5 Development, Health, and Disease Research Program, University of California, Irvine, Orange, CA USA; 6 NeuroCure Cluster of Excellence, Berlin, Germany

**Keywords:** Maltreatment, Affective control, Internalizing disorders, Resilience

## Abstract

Identifying cognitive-emotional mechanisms that distinguish risk from resilience for developing internalizing disorders after childhood maltreatment is essential for detecting early vulnerability and informing targeted interventions. We examined whether affective control and attentional biases contribute to internalizing symptoms versus resilience in children with maltreatment exposure during middle childhood. We recruited *N* = 208 children, aged 6–12 years, including *N* = 84 with maltreatment exposure from child welfare offices and clinics and *N* = 124 children without maltreatment exposure. An emotional flanker task was used to assess affective control and attentional biases. Associations between these cognitive-emotional mechanisms, internalizing symptoms, and trait resilience were examined. Children with maltreatment exposure showed reduced affective control and heightened attentional processing of distracting angry faces, indicating increased sensitivity to threat-related information. Among children exposed to maltreatment, greater avoidance of threat was associated with more internalizing symptoms. Notably, greater attentional processing of distracting happy faces was linked to higher trait resilience across both groups and moderated the association between maltreatment exposure and internalizing symptoms. These findings suggest that children with maltreatment exposure exhibit early difficulties in affective control and a bias toward negative information processing. Crucially, enhanced attentional engagement with positive stimuli may function as a protective factor, buffering against internalizing symptoms and fostering resilience. Early interventions targeting affective control and the processing of positive social cues may improve mental health outcomes in children exposed to maltreatment.

**Supplementary Information:**

The online version contains supplementary material available at https://doi.org/10.1007/s00787-026-03055-x.

## Introduction

Maltreatment during childhood is one of the most potent and pervasive risk factors for the development of internalizing disorders throughout the lifespan [1, 2]. Such risk may be in part attributable to changes in affective control and biases in attentional processing in terms of privileging negative information over positive information in the environment. Difficulties in affective control and biased information processing have been proposed as latent vulnerabilities that increase the likelihood of developing future mental health problems in children with maltreatment exposure [3]. Conversely, emerging evidence suggests that enhanced processing of positive emotional cues may potentially function as a protective mechanism, fostering resilient functioning even in the face of early adversity [4].

Affective control refers to the application of cognitive control to affective contexts, that is, the capacity to attend and respond to goal-relevant affective information, while inhibiting attention and responses toward distracting affective information [5]. Affective control has been suggested to represent a fundamental building block of emotion regulation. While evidence suggesting emotion regulation deficits in children with maltreatment exposure is accumulating [6–9], evidence on reduced affective control is still sparse. A few studies have reported affective control to be in part impaired in adults and adolescents with maltreatment exposure [10, 11]. Other studies have linked better affective control in adolescents with decreased internalizing symptoms and enhanced mental health [5].

Children with maltreatment exposure further tend to exhibit attentional biases in response to negative stimuli, similar to what has been observed in adult patients with depression and anxiety disorders [12]. Studies using dot-probe paradigms report that children with maltreatment exposure exhibit either vigilance toward or avoidance of threat, both of which have been linked to psychopathological symptoms [13–15].

In contrast to the well-documented attentional biases toward negative stimuli, emerging evidence suggests that attentional engagement with positive emotional stimuli, such as happy faces, may serve as a protective factor against internalizing disorders in children and adults [16–18]. However, findings regarding the processing of positive emotional stimuli in children with maltreatment exposure have been mixed. Several studies report reduced neural responsiveness to positive social stimuli, such as happy faces and peer acceptance feedback, with these patterns being associated with internalizing symptoms [19–21]. In the domain of self-generated thoughts, children and adolescents with maltreatment exposure report fewer positive thoughts, which might reflect increased depression risk [22, 23]. On the other hand, additional studies suggest a moderating role of processing of positive emotional stimuli. One study in children with maltreatment exposure demonstrated that children’s neural response to positive social stimuli is a longitudinal predictor of lower internalizing symptoms [4]. A recent study reported that heightened perceived intensity of happy faces in maltreatment exposed children and adolescents was associated with better mental health and more social support [24]. Taken together, these findings suggest that while maltreatment may impair positive social stimuli processing in some cases, heightened engagement with happy faces could also serve as a protective or compensatory mechanism. Specifically, the saliency of positive social stimuli might moderate the relationship between maltreatment exposure and internalizing symptoms, potentially buffering against adverse psychological outcomes.

In sum, alterations in affective control as well as biased attentional processing in children with maltreatment exposure might function as cognitive-emotional markers of risk and resilience after such exposures, specifically in the context of the development of internalizing disorders. One validated way to probe affective control as well as selective attention biases for both positive and negative information is the emotional flanker task [25–27]. In adults, performance on this task has been linked to depressive symptoms and rumination, reflecting difficulties in emotional conflict processing [28]. For example, Pe, et al. [25] reported that, when focusing on a negative target, trait ruminators ignored irrelevant positive information to a greater extent, showing a decreased interference effect. This suggests reduced attentional processing of irrelevant positive information among trait ruminators.

In the current study, we investigated affective control and attentional biases in a sample of children during middle childhood, with and without maltreatment exposure to potentially uncover neurocognitive markers of risk versus resilience in regard to the development of internalizing disorders. Middle childhood is a critical period for emerging mental health and behavioral outcomes that coincides with ongoing neurodevelopmental plasticity [29] and an increased importance of peer relationships. For example, social difficulties during this stage have been linked to later internalizing and other mental health problems [30, 31]. Importantly, rates of anxiety and depressive symptoms rise substantially during adolescence, suggesting that cognitive-emotional vulnerabilities may already be detectable in earlier developmental stages. Studying affective control and attentional biases during middle childhood may therefore allow for the identification of early vulnerability and resilience markers prior to the consolidation of symptom trajectories, thereby informing developmentally sensitive prevention efforts aimed at reducing later mental health risk.

To probe both risk and potential resilience markers in cognitive-affective processing, we employed an emotional flanker task involving both angry and happy faces [32], which allows quantifying affective control and attention biases in emotionally conflicting contexts. While prior research has emphasized heightened sensitivity to threat, this study uniquely examines the potential resilience-promoting role of attentional engagement with positive emotional stimuli. We first hypothesized that children with maltreatment exposure show decreased affective control, as evidenced by increased interference effects in error percentages by incongruent emotional face stimuli. Considering evidence of heightened sensitivity to threatening information [15], we expected that children with maltreatment exposure would show greater difficulty in affective control when reacting to happy face targets, while distracted by angry faces. Secondly, based on studies in adults with depression and studies on threat-biases in children with maltreatment exposure [14, 25, 26], we hypothesized that children with maltreatment exposure show attentional biases during emotional conflict processing in terms of reaction times, showing greater interference effects when reacting to happy face targets while ignoring angry faces, and a smaller interference effect when reacting to angry faces while ignoring happy faces. Thirdly, we expected reduced affective control and attentional biases are specifically associated with internalizing symptoms and resilience in children with maltreatment exposure. Lastly, we hypothesized that the saliency of positive emotional stimuli, reflected in attentional engagement with happy faces moderates the relationship between maltreatment exposure and internalizing symptoms. Specifically, greater engagement with happy faces was expected to buffer against the adverse effects of maltreatment, reducing internalizing symptoms.

## Methods

### Participants

We recruited 84 children with maltreatment exposure (Mean age = 8.86, SD = 1.50) from a broad range of local child welfare and protection services, including government offices for child welfare, family assistants and counselors, and agencies of the child welfare sector. We recruited 124 healthy children without maltreatment exposure (Mean age = 8.53, SD = 1.58) from the community via newspaper and internet advertisement. General exclusion criteria for all children included severe mental or physical disability (e.g., intellectual disability or severe neurological disorders), severe chronic illness (e.g., autoimmune or metabolic diseases), medication with impact on neural, endocrine, or immune systems (e.g., psychotropic or steroid medication), severe accidents, and an IQ < 70. For assignment to the group of children without maltreatment exposure, participants were screened to exclude maltreatment, exposure to violence, or other severe traumatic life events. This study was conducted as part of the Kids2Health project, a larger research program investigating mechanisms of biological embedding following early adversity and their implications for disease risk across development.

### Maltreatment exposure

To assess features of maltreatment, we used the Maternal Interview for the Classification of Maltreatment (MICM) for the parental report [33]. The interview was administered by trained clinicians and based on caregiver reports. Responses were coded according to the Maltreatment Classification System MCS; [34], which provides specific criteria for classifying and quantifying the occurrence and features of subtypes of maltreatment. The interview covers a range of subtypes, including emotional maltreatment (i.e. emotional abuse and/or emotional neglect), sexual abuse, physical neglect (i.e. failure to provide and/or lack of supervision), physical abuse, and moral, legal and/or educational maltreatment. For a more nuanced assessment of emotional maltreatment, a further distinction was added between emotional abuse and emotional neglect, as well as for witnessing domestic violence as also described elsewhere [35]. For each incidence of exposure, severity of maltreatment is rated on a 5-point scale ranging from mild (1) to severe or life-threatening (5). Maltreatment maximum severity across subtypes was computed for each child. Maltreatment maximum chronicity across subtypes was calculated as the ratio of developmental periods affected by maltreatment relative to the total lived developmental periods of the child (0–100%). The number of experienced maltreatment subtypes was also assessed (ranging from 1 to 6). Published severity cutoff scores were used to include children in the maltreatment group (emotional maltreatment ≥ 2, physical neglect ≥ 2, physical abuse ≥ 1, sexual abuse ≥ 1) [2]. Children not meeting these cutoff criteria were classified as non-maltreated.

### Emotional flanker task

**Fig. 1:**
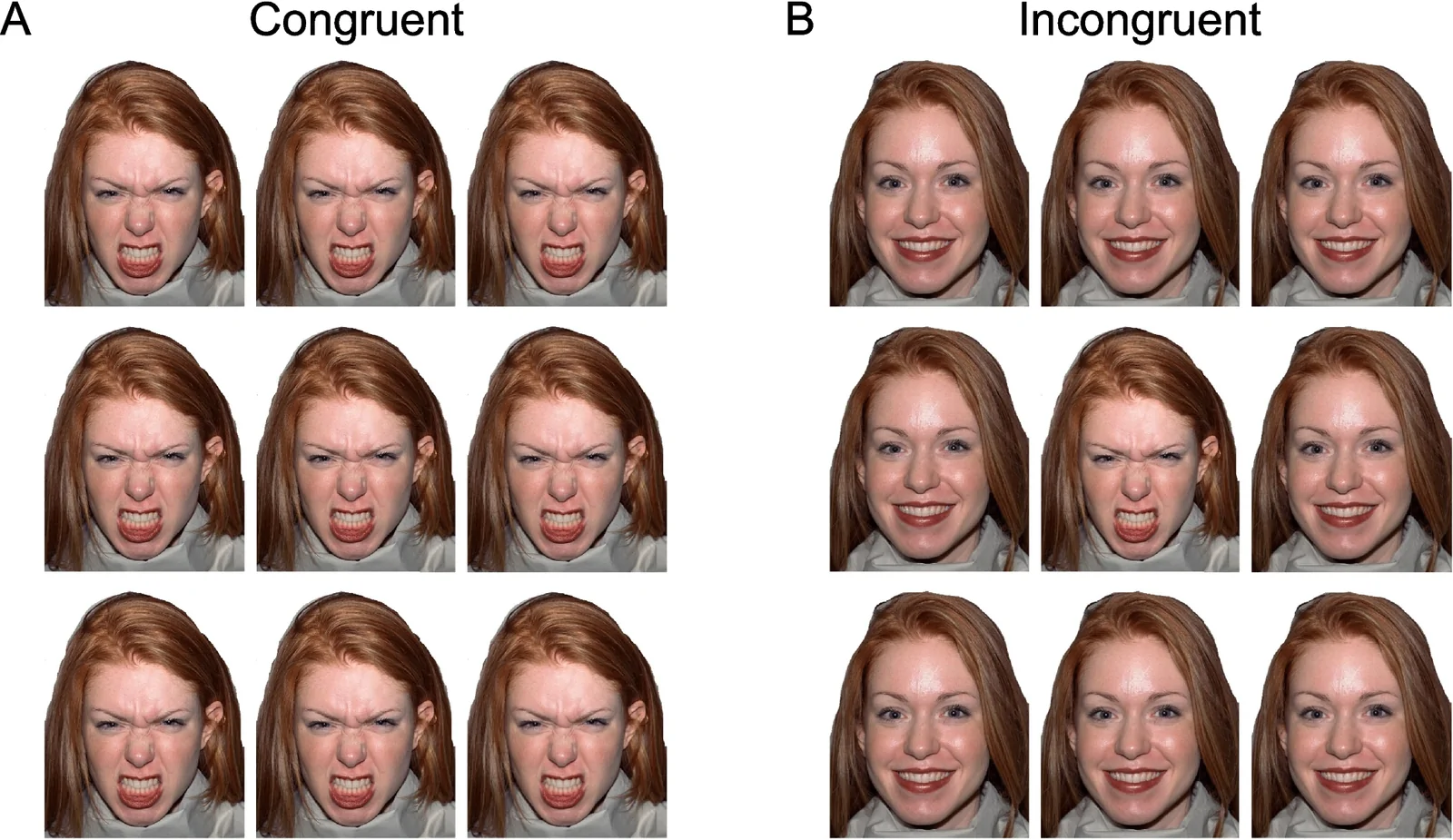
Example congruent (**A**; angry target with angry distractors) and incongruent (**B**; angry target with happy distractors) trials of the emotional flanker task. Participants indicated the emotion of the central target face while ignoring the flanking distractor faces. Interference effects were quantified as the difference between incongruent and congruent trials

To assess affective control an emotional flanker task (see Fig. 1) was used as in a previous developmental study [32]. Children had to respond as quickly as possible with their right index and middle fingers using the arrow keys on the keyboard to happy and angry target faces, pressing the left arrow key with the index finger for angry faces and the right arrow key with the middle finger for happy faces. Target faces (NimStim faces) were presented in the center of the screen. These target faces were flanked by eight distractor faces (picture size: 101 × 130 pixels) that were either all identical to or opposite in the emotion displayed relative to the target face. The distractor faces appeared directly around the target face at angles of 0°, 45°, 90°, 135°, 180°, 225°, 270°, and 315°, forming a 3 × 3 grid with the target face positioned in the center. A trial was considered congruent if the target face was flanked by identical faces portraying the same emotion and incongruent if the target face was flanked by identical faces portraying the opposite emotion. The eight distractor faces preceded the target face by 200 ms and remained on the screen together with the target until a response was given. Trials were always followed by a random intertrial interval (500, 1,000, or 1,500 ms). In total, there were 120 trials: 60 congruent trials and 60 incongruent trials (30 happy and 30 angry targets each). The task took approximately 7 min to complete. An increase in reaction times (RTs) on correct trials and error percentages (EPs) in incongruent compared to congruent trials indicated a so-called flanker congruency effect [36].

### Psychopathology and resilience measures

Dimensional psychopathology was measured with the Strength and Difficulties Questionnaire [SDQ] [37], as well as the Child Behavior Checklist [38], using parent report. The SDQ and the CBCL are widely used established instruments to measure externalizing and internalizing psychopathology symptoms with good validity and reliability [38, 39]. For the present analyses, internalizing and externalizing symptoms were operationalized as dimensional composite scores. Specifically, the CBCL broadband Internalizing Problems and Externalizing Problems scales and the SDQ internalizing and externalizing scores were first z-standardized within the total sample. The standardized CBCL and SDQ scores were then averaged separately to create unified internalizing and externalizing composite scores. To assess the presence of psychiatric diagnoses, parents completed the Kiddie Schedule for Affective Disorders and Schizophrenia – Present and Lifetime Version [K-SADS-PL;] [40], a structured clinical interview for diagnosing DSM-IV psychiatric diagnoses in children and adolescents. To measure trait resilience in children, parents completed the Ego Resiliency Scale that assesses how their children cope with stress and adversity [41, 42].

### Covariates

#### Socioeconomic status

Socioeconomic status (SES) was assessed based on the Winkler and Stolzenberg Index [43]. The multi-dimensional index score represents the sum (range 3–21) of three metric components, that is, education and occupational qualification, occupational status, and net income of the household (range 1–7 each).

#### Cognitive ability

Cognitive ability was assessed with the Snijders-Oomen Nonverbal Intelligence Test for children aged 2–8 years [SON-R 2–8] [44] and the Snijders-Oomen Nonverbal Intelligence Test for children and adults aged 6–40 years [SON-R] [6–40, 45]. The SON-R was chosen as a nonverbal measure of cognitive ability to minimize language- and education-related biases.

### Data preprocessing and analysis

Participants were excluded from analysis if they made more than 50% errors in the congruent trials, indicating insufficient task engagement, consistent with prior flanker studies employing performance-based exclusion criteria e.g. [46]. Participants were also excluded if their error rates exceeded 3 standard deviations above the group mean in any condition. For RT analyses, only correct trials were included. RTs shorter than 200 ms were removed, as well as RTs exceeding 3 standard deviations above each participant’s mean within each condition (Congruent Positive, Congruent Negative, Incongruent Positive, Incongruent Negative). Participants were additionally excluded if more than 10% of their trials were classified as RT outliers, consistent with participant-level exclusion procedures used in prior flanker studies e.g. [47]. In total, 4 children without maltreatment exposure and 7 children with maltreatment exposure were excluded. The final sample comprised 120 non-maltreated and 77 maltreated children. Subsequently, 2 (Target Emotion: Angry vs. Happy) × 2 (Congruency: Congruent vs. Incongruent) × 2 (Group: Maltreatment exposure (MT) vs. No maltreatment exposure (NMT)) ANCOVAs were performed on the error percentages and the RTs to investigate differences in interference effects. Age, sex, IQ and socio-economic status (SES) were used as covariates. Difference scores were computed to index interference effects per target emotion condition for error percentages and RTs. In the happy target condition, difference scores reflect interference from angry faces, whereas in the angry target condition, difference scores reflect interference from happy faces. Statistical analyses were conducted using SPSS (version 29), while data preprocessing and Figure generation were performed in R (version 4.3.2).

## Results

### Demographic features and sample characteristics

Table 1 presents the demographic features and sample characteristics. Children with maltreatment exposure showed significantly decreased cognitive ability, decreased trait resilience, and had lower SES, while showing increased internalizing and externalizing symptom scores, as well an increased number of psychiatric diagnoses compared to children without maltreatment exposure. The distribution of the most common psychiatric diagnosis categories across groups is provided in Table S1 (Supplementary Material).

**Table 1:** Demographic characteristics and sample features of the analysed sample of children with and without maltreatment exposure

		Children with maltreatment exposure (*N* = 77)	Non-Maltreated Children (*N* = 120)	
Measure		*Mean (SD)*	*Mean (SD)*	*p*
Age (years) IQ SES		8.88 (1.57)	8.54 (1.48)	0.13
		96.60 (15.71)	110.94 (15.148)	< 0.001
		10.07 (5.23)	17.58 (3.54)	< 0.001
		*n (%)*	*n (%)*	*p*
Sex (% female)		37 (48)	58 (48)	0.54
		*Mean (SD)*	*Mean (SD)*	*p*
Maltreatment maximum severity		3.18 (0.90)	0.06 (0.25)	< 0.001
Maltreatment maximum chronicity (%)		70.78 (27.57)	3.33 (14.63)	< 0.001
Internalizing symptom score		0.34 (0.93)	−0.25 (0.74)	< 0.001
Externalizing symptom score		0.50 (1.01)	−0.38 (0.59)	< 0.001
Number of psychiatric diagnoses		1.52 (1.22)	0.38 (0.79)	< 0.001
Ego Resiliency		5.91 (1.25)	6.99 (0.93)	< 0.001

### Emotional flanker task

#### Error percentages

**Fig. 2:**
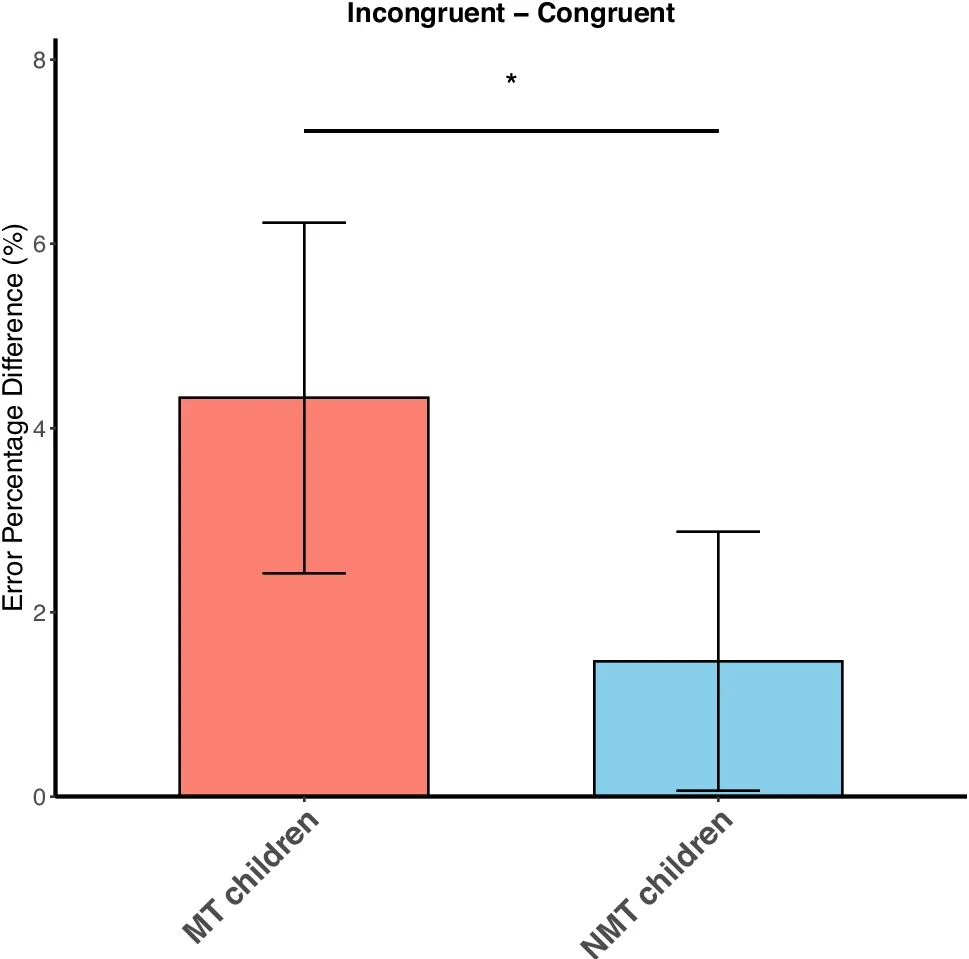
Children with maltreatment exposure exhibited greater difficulty with affective control, as indicated by a larger interference effect in error percentages (i.e., a greater difference between incongruent and congruent trials). *Note.* Estimated means and 95% confidence intervals are shown, controlling for age, sex, IQ, and SES

To test our primary hypothesis regarding reduced affective control in children with maltreatment exposure, we first examined the congruency × group interaction. There was a significant congruency × group interaction (*F*(1, 189) = 5.21, *p* = 0.024, *η*_p_^2^ = 0.03). Conducting an ANCOVA (controlled for age, sex, IQ and SES) on the difference score in total EPs, showed that children with maltreatment exposure exhibited a significantly greater interference effect, pointing to decreased affective control, (*F*(1, 189) = 4.42, *p* = 0.037, *η*_p_^2^ = 0.02) (Fig. 2). We further tested whether this effect was modulated by target emotion (target emotion × congruency × group interaction). This interaction was not significant (*F*(1, 189) = 0.78, *p* = 0.379, *η*_p_^2^ = 0.004), suggesting that the problems in affective control were independent of emotion condition. Additionally, there was a target emotion × congruency interaction, with a stronger interference effect when reacting to angry faces being distracted by happy faces (*F*(1, 189) = 6.17, *p* = 0.014, *η*_p_^2^ = 0.03). There were no further significant interactions or main effects (*Fs* > 2.35, *ps* > 0.127, see Table S2). As a sensitivity analysis, adding the number of psychiatric diagnoses as an additional covariate, the significant congruency × group interaction remained.

#### Reaction times

**Fig. 3:**
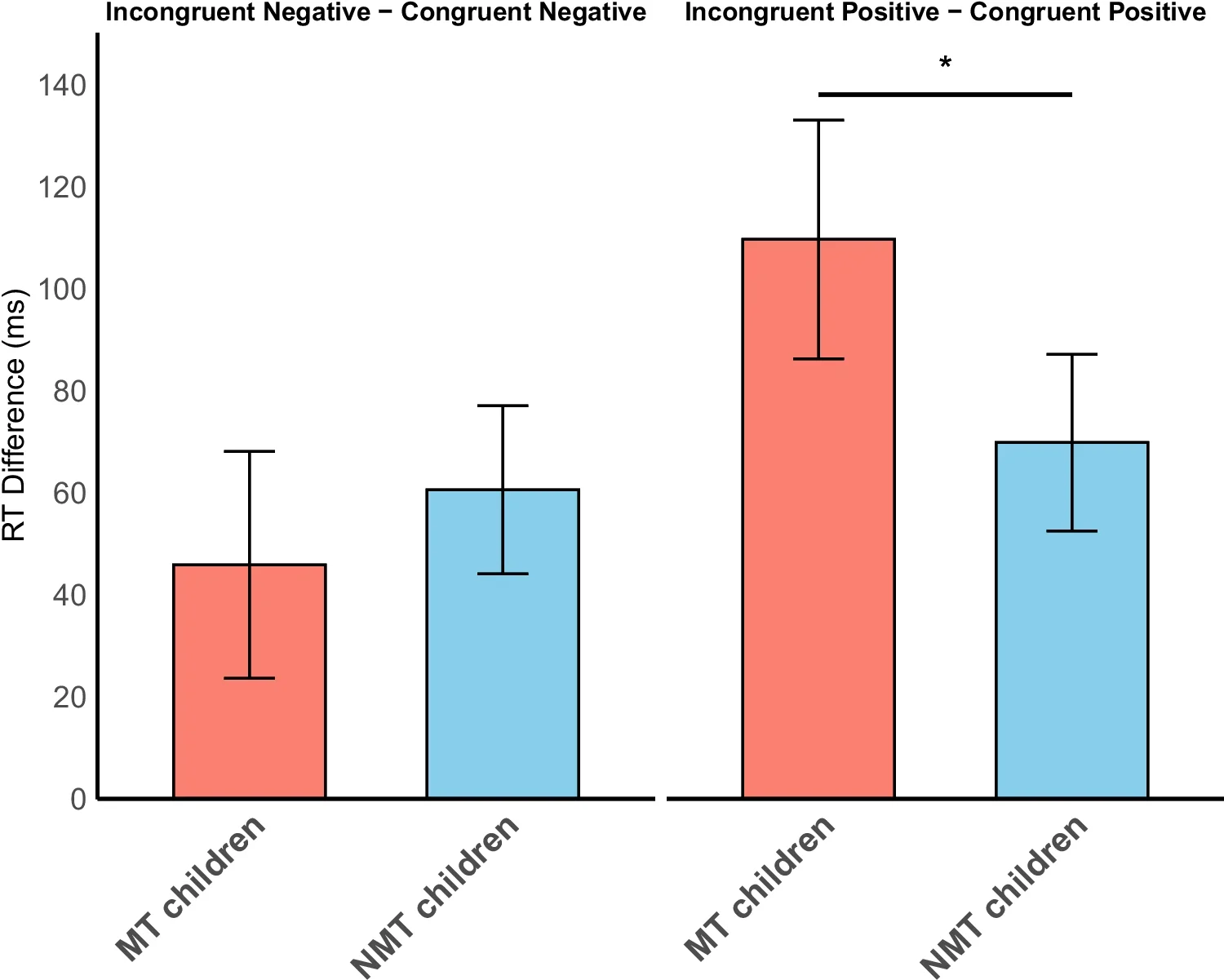
Children with maltreatment exposure exhibited heightened attentional processing of distracting angry faces, indicating increased sensitivity to threat-related information (i.e., a greater difference in reaction times between incongruent and congruent trials). *Note.* Estimated means and 95% confidence intervals are shown, controlling for age, sex, IQ, and SES

To test our primary hypothesis regarding emotion-specific attentional biases in children with maltreatment exposure, we examined the target emotion × congruency × group interaction. This interaction was significant *(F*(1, 189) = 5.27, *p* = 0.023, *η*_p_^2^ = 0.03). Conducting ANCOVAs (controlled for age, sex, IQ and SES) on the difference scores in RTs indicated that children with maltreatment exposure showed increased RTs when reacting to happy faces while ignoring angry faces suggesting heightened emotional conflict processing when distracted by threat-related information (*F*(1, 189) = 5.72, *p* = 0.018, *η*_p_^2^ = 0.03) (Fig. 3). There was no significant difference in emotional conflict processing when reacting to angry faces while ignoring happy faces (*F*(1, 189) = 0.86, *p* = 0.355, *η*_p_^2^ = 0.005). There were no further significant interactions (*Fs* < 1.42, *ps* > 0.234, see Table S2). There was a significant main effect of group, with children with maltreatment exposure showing slower RTs (*F*(1, 189) = 4.86, *p* = 0.029, *η*_p_^2^ = 0.03). Additionally, there was a main effect of congruency, suggesting an overall flanker interference effect on RTs for children, with slower RTs for incongruent conditions vs. congruent conditions (*F*(1, 189) = 23.10, *p* < 0.001, *η*_p_^2^ = 0.11). There were no further main effects (*Fs* < 1.51, *ps* > 0.221, see Table S2). As a sensitivity analysis, adding the number of psychiatric diagnoses as an additional covariate reduced the target emotion × congruency × group interaction to trend-level significance (*p* = 0.066). This suggests that the heightened emotional conflict processing in children with maltreatment exposure when distracted by threat-related information is largely independent of clinical status.

#### Associations of affective control and attentional biases with psychopathology symptoms and trait resilience

**Fig. 4:**
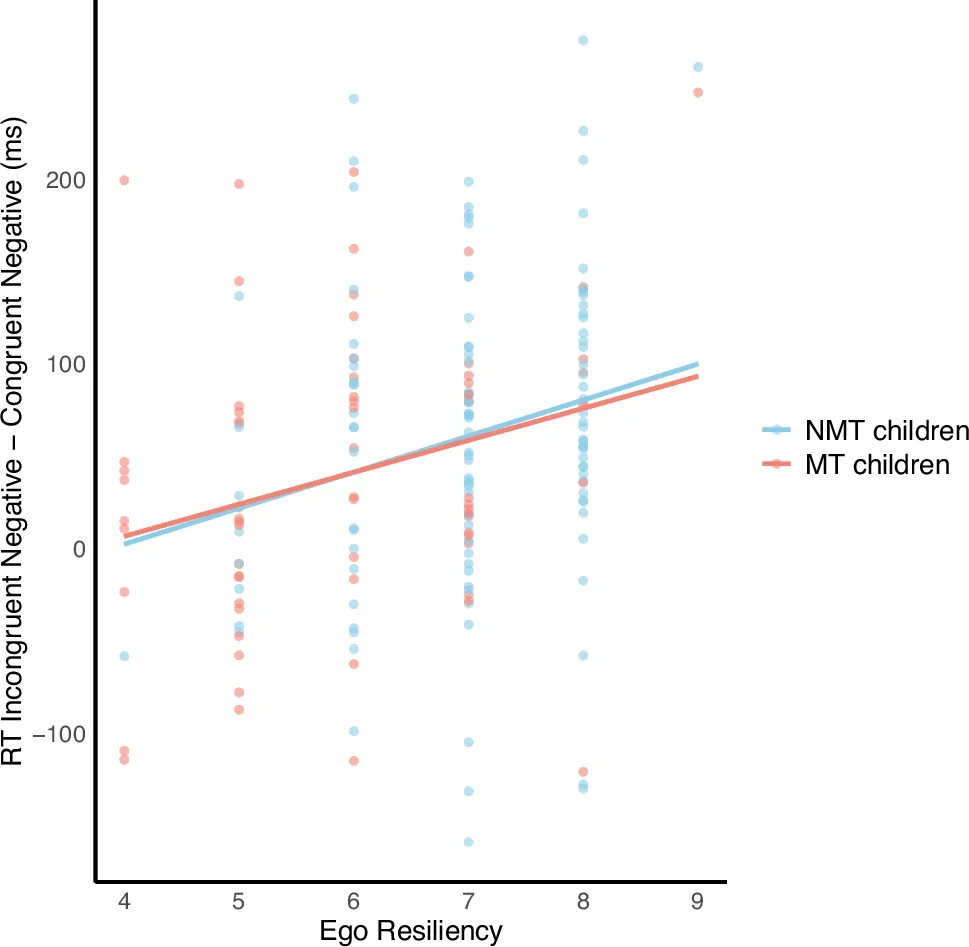
For children with maltreatment exposure (red) as well as children without maltreatment exposure (blue), greater attentional processing of distracting happy faces (i.e., larger reaction time differences between incongruent and congruent trials in the angry target condition) was associated with increased trait resilience

Pearson correlations were conducted to examine associations between attentional bias indices (RT difference scores), affective control (EP difference score), symptom dimensions, and trait resilience. These analyses were exploratory in nature. Complete correlation matrices for the maltreated group, the non-maltreated group, and the total sample are provided in Table S3 (Supplementary Material). Formal moderation analyses testing a priori hypotheses are reported separately. Within children with maltreatment exposure, a smaller interference effect when reacting to happy faces while ignoring angry faces was negatively associated with internalizing symptoms (r = − 0.26, *p* = 0.038), but not with externalizing symptoms (r = − 0.13, *p* = 0.301) or trait resilience (r = 0.17, *p* = 0.171). A smaller interference effect when reacting to angry faces while ignoring happy faces was significantly associated with internalizing symptoms (r = − 0.48, *p* < 0.001) and externalizing symptoms (r = − 0.33, *p* = 0.010). However, when controlling for the overlap between symptom dimensions using partial correlations, only the association with internalizing symptoms remained significant (r = − 0.38, *p* = 0.002), whereas the association with externalizing symptoms was no longer significant (r = − 0.06, *p* = 0.673). In the total sample, a smaller interference effect when reacting to angry faces while ignoring happy faces was also associated with internalizing symptoms (r = − 0.22, *p* = 0.003), but not with externalizing symptoms (r = − 0.12, *p* = 0.118). Regarding trait resilience, a greater interference effect when reacting to angry faces while ignoring happy faces was associated with higher resilience scores in both children with and without maltreatment exposure (r = 0.27, *p* = 0.027; r = 0.23, *p* = 0.012; see Fig. 4). No associations were observed between error-based affective control (difference score in error percentages) and psychopathology symptoms or trait resilience (r < 0.14, ps > 0.275).

#### Attentional processing of distracting happy faces moderates the relationship between maltreatment exposure and internalizing symptoms

**Fig. 5:**
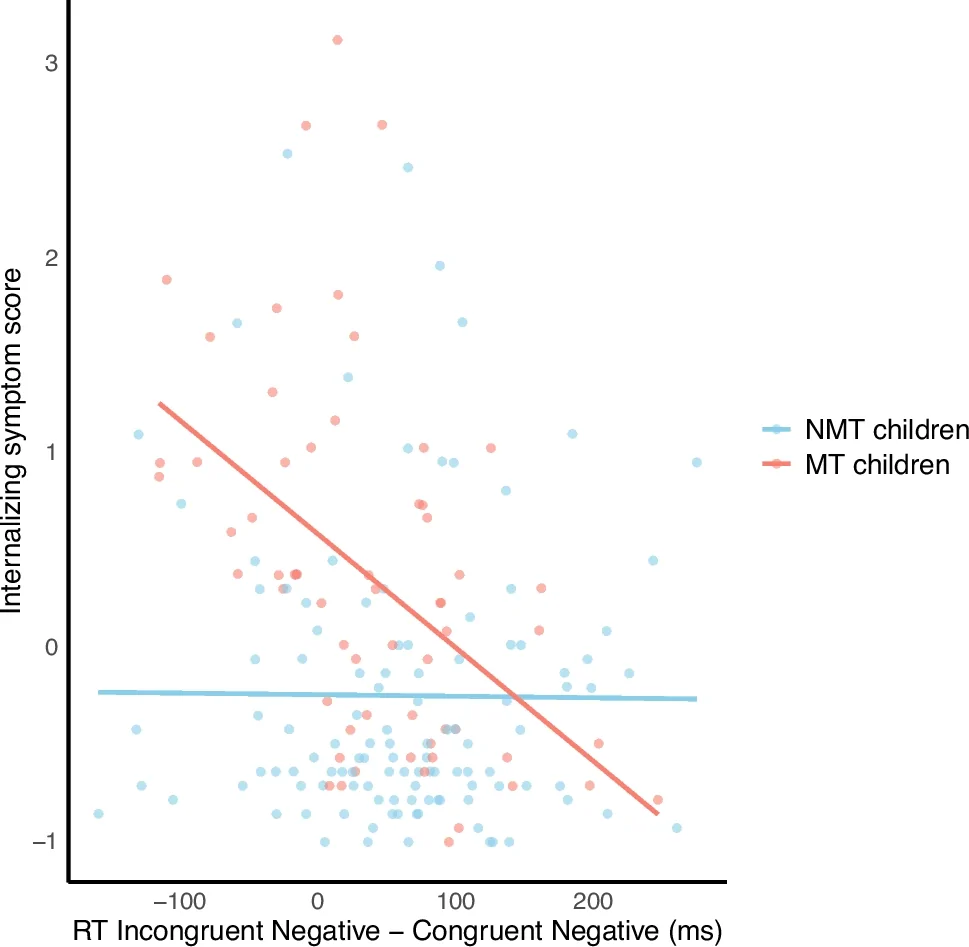
Attentional processing of distracting happy faces (i.e., larger reaction time differences between incongruent and congruent trials in the happy distractor condition) moderated the association between maltreatment exposure and internalizing symptoms. Specifically, among children with maltreatment exposure, greater processing of happy face distractors was associated with fewer internalizing symptoms

Given our a priori hypothesis focusing on internalizing psychopathology, we examined the potential moderating role of the interference effect in the angry target condition (i.e., when reacting to angry faces while ignoring happy faces) on the relationship between maltreatment exposure and internalizing symptoms. An ANCOVA was conducted with internalizing symptoms as the dependent variable. The interference effect (Incongruent Negative – Congruent Negative) and group factor (No maltreatment exposure vs. Maltreatment exposure) were used as predictors, while SES, age, sex and IQ were used as additional covariates. There was a main effect of the interference effect (*F*(1, 167) = 14.13, *p* < 0.001, *η*_p_^2^ = 0.08) and group (*F*(1, 167) = 11.12, *p* < 0.001, *η*_p_^2^ = 0.06). Crucially, a significant RT difference score x group interaction was observed (*F*(1, 167) = 14.29, *p* < 0.001, *η*_p_^2^ = 0.08), indicating that the interference effect in the angry target condition moderated the relationship between maltreatment exposure and internalizing symptoms (Fig. 5). More specifically, greater interference effects in this condition (reflecting attentional engagement with distracting happy faces) attenuated internalizing symptoms in children with maltreatment exposure.

## Discussion

In this study, we investigated affective control and attentional biases in a large sample of children in middle childhood, with and without maltreatment exposure, to uncover cognitive-emotional markers of risk and resilience during childhood in relation to internalizing symptoms. Affective control as well as attentional biases towards negative and away from positive stimuli in the environment might serve as markers for disease risk versus resilience following maltreatment exposure that could be targeted by early interventions. For this purpose, we used an emotional flanker task with angry and happy face stimuli, to scrutinize affective control mechanisms and attentional biases to negative and positive social stimuli.

As hypothesized, children with maltreatment exposure exhibited overall decreased affective control in terms of decreased emotional conflict resolution on the flanker task, irrespective of the emotional condition. Because these congruency-related effects on error rates were not modulated by emotion, they could potentially be partly explained by more general differences in executive control. However, prior literature suggests that attentional control of affective stimuli shows only partial overlap with control of non-affective stimuli [5, 48]. Importantly, these effects remained significant after controlling for clinical diagnoses (e.g., internalizing disorders) and other confounders, indicating that the observed alterations in affective control are not solely attributable to current clinical status, but may reflect an underlying neurocognitive marker of risk. This is in line with studies in adults and adolescents showing that maltreatment exposure is associated with reduced affective control [11], indicating that such problems in affective control already emerge in middle childhood. Because affective control has been considered to be an important cognitive prerequisite for successful emotion regulation, reduced affective control likely further influences more elaborate emotion regulation capacities throughout development, thereby increasing the risk for developing mental disorders in children exposed to maltreatment.

Additionally, children with maltreatment exposure showed attentional biases during emotional conflict processing. Children with maltreatment exposure exhibited a greater interference when reacting to happy face targets while ignoring angry faces. This finding is in line with studies in adults with and without depression, in which depression symptoms and rumination were associated with greater attentional processing of distracting angry faces during emotional conflict processing [25]. Furthermore, these findings are in accordance with studies reporting increased attentional biases towards threat-related information in children and adolescents with maltreatment exposure e.g. [15]. Interestingly however, exclusively internalizing symptoms in children with maltreatment exposure, as assessed with a compositive internalizing symptom score, were associated with less attentional processing of distracting angry faces, possibly indicating a threat avoidance in these children. Thus, although children with maltreatment exposure exhibited heightened threat-related attentional engagement compared to controls, within this group lower levels of attentional processing of distracting angry faces were associated with higher internalizing symptoms. This pattern could suggest that a certain degree of threat sensitivity may be functionally adaptive in adverse environments, whereas reduced engagement with threat cues may reflect avoidance or dysregulated processing in children with maltreatment exposure in middle childhood.

Some studies using dot-probe paradigms have indeed described an attention bias away from threat in children with maltreatment exposure in middle childhood and early adolescence, which was associated with psychopathological symptoms and social functioning [13, 14]. It has been suggested that such threat avoidance might represent a short-term strategy to attenuate the impact of threat cues when other means of emotion regulation are not available [49], but in the long-term might lead to a failure to habituate to threat cues [13]. In the literature, maltreatment has been associated with both attentional engagement toward threat and attentional avoidance. Against this background, the heterogeneity in findings may reflect differences in study design, task paradigms, and sample characteristics across studies. Longitudinal studies are needed to clarify how threat-related attentional processing following maltreatment exposure relates to psychopathology.

Children with maltreatment exposure did not show a smaller interference effect, when reacting to angry faces while ignoring happy faces, when compared to children without maltreatment exposure, suggesting, in part, normal attentional processing of positive stimuli in the environment. Attentional processing of distracting happy faces, however, was associated with trait resilience in both groups, similarly to what has been reported in healthy adults using the dot-probe task [16]. As hypothesized, attentional processing of distracting happy faces moderated the relationship between maltreatment exposure and internalizing symptoms, with greater engagement with happy faces buffering against the development of internalizing symptoms. This finding supports the proposed protective role of positive emotional stimuli, highlighting its potential as a resilience-promoting mechanism in children with maltreatment exposure. These results are thus in accordance with findings that suggest that increased saliency of positive affective cues might be particularly protective against developing internalizing disorders promoting resilient functioning [4, 16]. Furthermore, our findings suggest that attentional processing of positive social cues could represent an early-stage resilience mechanism, preceding higher-order processes such as cognitive reappraisal. In line with developmental and neurocognitive models of resilience [50, 51], such early filtering mechanisms may help children with maltreatment exposure maintain sensitivity to positively-valenced social information despite emotionally challenging contexts and may shape subsequent appraisal and reappraisal processes. In addition, heightened saliency of positive social cues may promote prosocial and affiliative tendencies that support the development and maintenance of supportive relationships over time, consistent with recent findings showing that greater perceived intensity of positive facial expressions was associated with reduced symptomatology and higher perceived social support following childhood adversity [24].

From a clinical perspective, these findings are critical and deliver important targets for the development of early interventions in children who experienced maltreatment. Our results suggest that early interventions should not only be aimed at modifying attentional biases in relation to negative information but should also aim to strengthen attentional processing of positive information, which might be useful to build resilience and mitigating risk for internalizing disorders. Cognitive Bias Modification (CBM) trainings focusing on threat-related information have shown some success in reducing affective symptoms in children and adolescents [52] and may represent a promising avenue for developmentally adapted interventions targeting positive information processing. In addition, cognitive trainings designed to improve affective control in adolescents [53] could potentially be adapted for children in middle childhood in a developmentally appropriate manner. Therapeutic interventions on the individual and systemic level that aim at tackling cognitive biases and at cultivating positive affect could be suitable, as such interventions have been shown to be effective with depressed patients [54, 55].

A number of limitations should be noted. First, the present study was cross-sectional in design, precluding any claims about causal mechanisms and thus pointing to the need for further longitudinal investigations. Future longitudinal studies could confirm whether alterations in affective control and attentional biases—particularly reduced engagement with positive emotional stimuli—serve as neurocognitive markers of risk or resilience. Such research would be crucial for validating these mechanisms as targets for early intervention. Secondly, an additional measure of state resilience could be helpful to further strengthen and specify the link between resilience and increased attentional processing of positive stimuli in the environment.

## Conclusion

This study provides evidence that already in middle childhood, children with maltreatment exposure show reduced affective control and heightened attentional processing of negative social information. At the same time, increased attentional engagement with positive emotional cues, such as happy faces, emerged as a potential protective factor—moderating the impact of early adversity on internalizing symptoms. These results identify early attentional processes as promising targets for resilience-promoting interventions. Strengthening affective control and enhancing the salience of positive emotional stimuli could be key strategies in improving mental health outcomes in children with maltreatment exposure.

## Supplementary Information

Below is the link to the electronic supplementary material.

Supplementary file1 (DOCX 28 KB)

## Data Availability

The data that support the findings of this study are available upon request from the corresponding author.
