# Supplementary material for: Seeing the positive in the face of adversity: neurocognitive markers of risk and resilience in emotional conflict processing in children with maltreatment exposure
Source: Eur Child Adolesc Psychiatry. 2026 May 18;35(9):2881–91. doi: 10.1007/s00787-026-03055-x (PMC13695692; doi:10.1007/s00787-026-03055-x)
Supplement: Supplementary file 1 [file 787_2026_3055_MOESM1_ESM.docx]

# Supplementary Material

**Seeing the Positive in the Face of Adversity: Neurocognitive Markers of Risk and Resilience in Emotional Conflict Processing in Children With Maltreatment Exposure**

Ferdinand Hoffmann^1,2,3^, Lea Bentz^1,4^, Sonja Entringer^1,2,5^, Sibylle M. Winter^2,4^, Claudia Buss^1,2,5^, and Christine Heim^1,2,6*^

^1^Charité – Universitätsmedizin Berlin, corporate member of Freie Universität Berlin and Humboldt-Universität zu Berlin, Institute of Medical Psychology, Berlin, Germany; ^2^Division of Clinical-Psychological Intervention, Department of Education and Psychology, Freie Universität Berlin, Berlin, Germany; ^3^German Center for Mental Health (DZPG), partner site Berlin-Potsdam, Germany; ^4^Charité – Universitätsmedizin Berlin, corporate member of Freie Universität Berlin and Humboldt-Universität zu Berlin, Department of Child and Adolescent Psychiatry, Psychotherapy and Psychosomatics, Berlin, Germany; ^5^Development, Health, and Disease Research Program, University of California, Irvine, Orange, CA, USA; ^6^NeuroCure Cluster of Excellence, Berlin, Germany.

**Table S1** The distribution of the most common psychiatric diagnosis categories across groups

| **Diagnosis category** | **Children with Maltreatment Exposure (n = 77)** | **Children without Maltreatment Exposure (n = 120)** |
| --- | --- | --- |
| Any psychiatric diagnosis | 59 (76.6%) | 29 (24.2%) |
| Depressive disorders | 10 (13.0%) | 5 (4.2%) |
| Anxiety disorders | 10 (13.0%) | 6 (5.0%) |
| ADHD | 33 (42.9%) | 8 (6.7%) |
| ODD/CD | 14 (18.2%) | 2 (1.7%) |
| PTSD | 12 (15.6%) | 0 (0.0%) |

*Note.* Values are n (%). Categories reflect children with at least one diagnosis within the respective category; children may meet criteria for more than one category. ADHD = Attention-Deficit/Hyperactivity Disorder, ODD = Oppositional Defiant Disorder, CD = Conduct Disorder, PTSD = Posttraumatic Stress Disorder.

**Table S2** Full factorial ANCOVA results for error percentages and reaction times

| Effect | F(1,189) | p | ηp² |
| --- | --- | --- | --- |
| **Error Percentages** |  |  |  |
| Target Emotion | 0.59 | .443 | .003 |
| Congruency | 2.35 | .127 | .012 |
| Group | 1.65 | .201 | .009 |
| Congruency × Group | 5.21 | .024 | .027 |
| Target Emotion × Congruency | 6.17 | .014 | .032 |
| Target Emotion × Group | <0.01 | .972 | <.001 |
| Target Emotion × Congruency × Group | 0.78 | .379 | .004 |
| **Reaction Times** |  |  |  |
| Target Emotion | 1.51 | .221 | .008 |
| Congruency | 23.10 | < .001 | .109 |
| Group | 4.86 | .029 | .025 |
| Congruency × Group | 1.28 | .259 | .007 |
| Target Emotion × Congruency | 0.82 | .775 | <.001 |
| Target Emotion × Group | 1.12 | .292 | .007 |
| Target Emotion × Congruency × Group | 5.27 | .023 | .027 |

*Note.* All analyses controlled for age, sex, IQ, and socio-economic status (SES).

**Table S3** Associations of affective control and attentional biases with psychopathology symptoms and trait resilience

| **Total Sample** | 1 | 2 | 3 | 4 | 5 | 6 |
| --- | --- | --- | --- | --- | --- | --- |
| 1. RT Difference Score (Happy Target / Angry Distractor) | — |  |  |  |  |  |
| 2. RT Difference Score (Angry Target / Happy Distractor) | -.05 | — |  |  |  |  |
| 3. Error Difference Score | .26*** | -.04 | — |  |  |  |
| 4. Internalizing Symptoms | .00 | -.22** | -.06 | — |  |  |
| 5. Externalizing Symptoms | .06 | -.12 | .09 | .60*** | — |  |
| 6. Trait Resilience | -.02 | .27*** | -.07 | -.65*** | -.62*** | — |
| **Children with Maltreatment Exposure** |  |  |  |  |  |  |
| 1. RT Difference Score (Happy Target / Angry Distractor) | — |  |  |  |  |  |
| 2. RT Difference Score (Angry Target / Happy Distractor) | -.03 | — |  |  |  |  |
| 3. Error Difference Score | .52*** | .11 | — |  |  |  |
| 4. Internalizing Symptoms | -.26* | -.48*** | -.14 | — |  |  |
| 5. Externalizing Symptoms | -.13 | -.33** | .10 | .59*** | — |  |
| 6. Trait Resilience | .17 | .27* | -.02 | -.62*** | -.63*** | — |
| **Children without Maltreatment Exposure** |  |  |  |  |  |  |
| 1. RT Difference Score (Happy Target / Angry Distractor) | — |  |  |  |  |  |
| 2. RT Difference Score (Angry Target / Happy Distractor) | -.05 | — |  |  |  |  |
| 3. Error Difference Score | .01 | -.12 | — |  |  |  |
| 4. Internalizing Symptoms | .10 | -.01 | -.09 | — |  |  |
| 5. Externalizing Symptoms | .11 | .14 | -.05 | .48*** | — |  |
| 6. Trait Resilience | -.04 | .23* | .03 | -.58*** | -.42*** | — |

*Note.* RT Difference Scores reflect reaction time differences between incongruent and congruent trials within each target emotion condition. Error Difference Score reflects the congruency effect in error percentages. Values represent Pearson correlation coefficients. *p < .05; **p < .01; ***p < .001.
